# The Significance of Overvaluation of Shape and Weight in Binge Eating Disorder

**DOI:** 10.1016/j.brat.2009.10.008

**Published:** 2009-10-24

**Authors:** Andrea B. Goldschmidt, Anja Hilbert, Jamie L. Manwaring, Denise E. Wilfley, Kathleen M. Pike, Christopher G. Fairburn, Ruth H. Striegel-Moore

**Affiliations:** a Department of Psychology, Washington University, 660 South Euclid Avenue, Campus Box 8134, St. Louis, MO, 63110, USA; goldscha@psychiatry.wustl.edu (Ms. Goldschmidt); manwaringj@psychiatry.wustl.edu (Ms. Manwaring); b Department of Psychology, Philipps University of Marburg, Gutenbergstrasse 18, Marburg, Germany; hilbert@staff.uni-marburg.de; c Department of Psychiatry, Washington University School of Medicine, 660 South Euclid Avenue, Box 8134, St. Louis, MO, 63110, USA; wilfleyd@psychiatry.wustl.edu; d Department of Psychology, Temple University Japan Campus, Azabu Hall 6th Floor, Minami-Azabu 2-8-12, Minato-ku, Tokyo, Japan, 106-0047; pike@tuj.ac.jp; e Department of Psychiatry, Oxford University, Warneford Hospital, Oxford OX3 7JX, England; credo@medsci.ox.ac.uk; f Department of Psychology, Wesleyan University, 207 High Street, Middletown, CT 06459, USA; rstriegel@wesleyan.edu

**Keywords:** Binge eating disorder, overvaluation of shape and weight, weight and shape concerns, classification

## Abstract

As publication of DSM-V draws near, research is needed to validate the diagnostic scheme for binge eating disorder (BED). Shape and weight overvaluation has stimulated considerable debate in this regard, given associations with psychosocial impairment and poor treatment outcome in BED. This study sought to further explore the convergent validity and diagnostic specificity of shape and weight overvaluation in BED. A total of 160 women with BED, and 108 women with non-eating disordered psychiatric disorders were recruited from the community. Women with BED were classified as more or less severe based on a global measure of eating-related psychopathology; subsequent receiver operating characteristics analysis determined that a threshold of at least “moderate” overvaluation best predicted membership into a more severe group. BED participants with threshold overvaluation exhibited poorer psychosocial functioning than those with subthreshold overvaluation, as well as participants with other psychiatric disorders. Discriminant function analysis revealed that threshold overvaluation predicted a diagnosis of BED versus other psychiatric disorder with 67.7% accuracy. Results suggest that shape and weight overvaluation is a useful diagnostic specifier in BED. Continued research is warranted to examine its predictive validity in natural course and treatment outcome studies.

Binge eating disorder (BED) is currently included in the DSM-IV-TR as a provisional diagnosis requiring further study (American Psychiatric Association, 2000). With the impending publication of DSM-V, several questions regarding the validity of BED and its diagnostic criteria remain (Latner & Clyne, 2008; Wilfley, Bishop, Wilson, & Agras, 2007; Wonderlich, Gordon, Mitchell, Crosby, & Engel, in press). In particular, it has been suggested that overvaluation of shape and weight be included in BED's diagnostic scheme in DSM-V, given evidence that it reliably predicts elevated levels of psychosocial impairment (Grilo et al., 2009; Grilo et al., 2008; Grilo, Masheb, & White, in press; Hrabosky, Masheb, White, & Grilo, 2007; Latner & Clyne, 2008; Mond, Hay, Rodgers, & Owen, 2007). Further research is needed to establish the clinical utility of this construct in adults with BED.

Overvaluation of shape and weight denotes the undue importance of shape and weight in one's scheme for self-evaluation (Fairburn, 2008). According to schema theory (Waller, Ohanian, Meyer, & Osman, 2000) and the cognitive behavioral model of eating disorders (Fairburn, 2008), shape and weight overvaluation refers to higher-order cognitive content reflecting core negative beliefs about the self (e.g., low self-esteem) that may manifest itself through automatic negative thoughts or dysfunctional assumptions regarding shape and weight. In contrast to body dissatisfaction, which may be contingent upon mood or current body size, and shape and weight concerns, which broadly encompass many aspects of shape- and weight-related attitudes, overvaluation of shape and weight represents a stable construct that is resistant to change (P. J. Cooper & Fairburn, 1993; Fairburn, 2008). Indeed, shape and weight overvaluation appears to be more closely related to changes in self-esteem over time, as compared to fluctuations in depressive symptoms (P. J. Cooper & Fairburn, 1993; Masheb & Grilo, 2003), and is at least partially responsible for persistence in bulimic symptoms over time (Fairburn, Stice et al., 2003). Given evidence that it is typically present in individuals with eating disorders, regardless of diagnostic group (Fairburn, Cooper, & Shafran, 2003), and appears to be of critical importance in maintaining these disorders (Fairburn, Peveler, Jones, Hope, & Doll, 1993; Fairburn, Stice et al., 2003), shape and weight overvaluation is considered by some, but not all (Slade, 1982; Waller, 2008), investigators to mark the “core psychopathology” of eating disorders (P. J. Cooper & Fairburn, 1993; Fairburn, 2008; Fairburn & Garner, 1986). As such, shape and weight overvaluation is currently a diagnostic criterion for both AN (i.e., “undue influence of body shape and weight on self-evaluation”) and BN (i.e., “self evaluation [that is] unduly influence by body shape and weight”; American Psychiatric Association, 2000).

While recognized as a feature of BN even before the publication of DSM-III (Russell, 1979), it was not until DSM-III-R that a construct approximating overvaluation of shape and weight (i.e., “persistent overconcern with body shape and weight”) was included as a diagnostic criterion for BN (American Psychiatric Association, 1987). DSM-IV's later refinement of this criterion to the more stringent overvaluation of shape and weight criterion purportedly reflects that the “critical disturbance is the undue influence of body shape and weight *on self-esteem*” (Walsh, 1992). Indeed, this distinction is supported by evidence that overvaluation of shape and weight discriminates individuals with eating disorders from healthy controls (Goldfein, Walsh, & Midlarsky, 2000; McFarlane, McCabe, Jarry, Olmsted, & Polivy, 2001), whereas body dissatisfaction and shape and weight concerns are less discriminating (Garfinkel et al., 1992; Hadigan & Walsh, 1991).

Although BED is a relatively new diagnostic entity, a great deal of empirical work has already focused on the nature of body image disturbance in BED. Individuals with BED report levels of shape and weight concerns that are commensurate to individuals with AN and BN, and significantly higher than both normal-weight and overweight individuals without eating disorders (Eldredge & Agras, 1996; Masheb & Grilo, 2000; Striegel-Moore et al., 2001; Striegel-Moore, Dohm et al., 2000; Wilfley, Schwartz, Spurrell, & Fairburn, 1997). These findings have stimulated research into the utility of including overvaluation of shape and weight in the diagnostic scheme for BED, either as an individual criterion or as a diagnostic specifier (i.e., a sub-category within a diagnosis that assists with treatment matching and/or prediction of treatment outcome). Several studies have documented that overvaluation of shape and weight among individuals with BED is associated with increased psychosocial impairment, including eating-related and general psychopathology, functional impairments, and decrements in quality of life (Grilo et al., 2009; Grilo et al., 2008; Grilo et al., in press; Hrabosky et al., 2007; Mond et al., 2007), as well as treatment-seeking behavior and poorer treatment response on some measures of outcome (Masheb & Grilo, 2008). Taken together, these findings suggest that overvaluation of shape and weight is a clinically relevant construct associated with elevated impairment and distress in BED.

According to research convention, overvaluation of shape and weight is considered to be clinically significant when shape and weight are at least moderately important in one's scheme for self-evaluation (Fairburn & Cooper, 1993). However, no research to date has validated the use of this threshold value, relative to other threshold values, among individuals with eating disorders. Several studies have demonstrated that individuals with full-syndrome and subclinical eating disorders are indistinguishable on measures of impairment and distress (Crow, Agras, Halmi, Mitchell, & Kraemer, 2002; Fairburn et al., 2007; Striegel-Moore, Dohm et al., 2000; Striegel-Moore, Wilson, Wilfley, Elder, & Brownell, 1998); expounding on these findings, it is possible that even less extreme overvaluation of shape and weight may nevertheless be associated with psychopathology and decrements in quality of life. If shape and weight overvaluation are to be included among BED's diagnostic criteria, it will be necessary to establish a threshold rating on this construct that is clinically meaningful and provides useful diagnostic information.

The purpose of the current study is to further examine the utility of including overvaluation of shape and weight in the diagnostic scheme for BED. Specific aims are to: 1) determine a threshold value of shape and weight overvaluation that is predictive of a more severe psychological profile in BED; 2) compare BED participants with threshold shape and weight overvaluation, BED participants with subthreshold overvaluation, and participants with other psychiatric disorders on measures of psychosocial and interpersonal functioning, and health care usage; and 3) examine how well the threshold value discriminates between women with BED and those with other psychiatric disorders.

## Method

### Participants

Participants were 268 Caucasian or African-American women (69.8% Caucasian, 30.2% African-American), aged 18-40 (*M* = 30.61; *SD* = 6.16). Participants were recruited from Connecticut, the Boston area, New York City, and Los Angeles to participate in the New England Women's Health Project (Striegel-Moore, Wilfley, Pike, Dohm, & Fairburn, 2000), a community-based study examining risk factors for BED. The sample consisted of 160 women diagnosed with BED, and 108 women diagnosed with a psychiatric disorder other than an eating disorder (psychiatric controls; PC). Eight participants (4 from the BED group and 4 from the PC group) did not respond to questionnaire items assessing overvaluation of shape and weight, and thus were excluded from all analyses. The final sample included 156 women with BED, and 104 PC women. For full sample characteristics, see Table 1.

### Procedures

Participants were recruited through community and media advertisements, and the use of consumer databases. Individuals interested in participating were administered a brief telephone screen and those who met basic eligibility criteria (i.e., age between 18 and 40; absence of medical conditions influencing eating behavior or body weight; absence of a psychotic disorder; being female, of black or white race, and born in the United States) were invited to complete an in-person assessment. Written informed consent was obtained from all participants. The study was approved by the IRBs at Wesleyan University and Columbia University. Detailed descriptions of recruitment and screening procedures are provided elsewhere (Pike, Dohm, Striegel-Moore, Wilfley, & Fairburn, 2001; Striegel-Moore, Dohm, Pike, Wilfley, & Fairburn, 2002; Striegel-Moore et al., 2005).

### Measures

#### Structured Clinical Interview for DSM-IV Axis I Disorders

All participants were given the Structured Clinical Interview for DSM-IV Axis I Disorders (SCID; First, Spitzer, Gibbon, & Williams, 1997) to ascertain psychiatric diagnoses. The SCID (First et al., 1997) is a well-established semi-structured interview assessing the full range of psychiatric disorders. Presence of a comorbid SCID diagnosis was used as a validator in analyses comparing women with BED reporting threshold and subthreshold overvaluation.

#### Eating Disorder Examination

Participants meeting diagnostic criteria for BED based on the SCID were given an abbreviated diagnostic version of the Eating Disorder Examination (EDE; Fairburn & Cooper, 1993) to confirm the diagnosis. The EDE is a semi-structured, interviewer-based instrument with established reliability and validity (Z. Cooper, Cooper, & Fairburn, 1989; Grilo, Masheb, Lozano-Blanco, & Barry, 2004; Rizvi, Peterson, Crow, & Agras, 2000; Rosen, Vara, Wendt, & Leitenberg, 1990). EDE items assessing weekly frequency of binge eating episodes (i.e., consumption of an unambiguously large amount of food accompanied by loss of control over eating) and binge eating-related distress over the past six months were used as validators in comparisons of women with BED reporting threshold and subthreshold overvaluation.

#### Eating Disorder Examination-Questionnaire

For the assessment of eating disorder psychopathology, all participants completed the Eating Disorder Examination-Questionnaire (EDE-Q; Fairburn & Beglin, 1994). The EDE-Q is a self-report questionnaire version of the EDE which generates a global index of eating-related pathology (including items measuring restraint, eating concern, weight concern, and shape concern). The EDE-Q was used to measure the independent variable of shape and weight overvaluation. For each participant, individual items assessing overvaluation of shape and overvaluation of weight (i.e., “Over the past four weeks, how much has your shape/weight influenced how you think about (judge) yourself as a person?”) were averaged to form a composite “overvaluation of shape and weight” item; responses ranged from 0 (not at all) to 6 (markedly). The EDE-Q global severity index was used to derive more and less severe BED groups for the receiver operating characteristics analysis. The EDE-Q subscales have demonstrated adequate internal consistency and retest reliability (Luce & Crowther, 1999; Mond, Hay, Rodgers, Owen, & Beumont, 2004). The measure has also shown convergent validity with the EDE across both eating disordered and non-eating disordered samples in the measurement of eating-related attitudes, although the EDE-Q tends to produce higher ratings across subscales than the EDE (Black & Wilson, 1996; Fairburn & Beglin, 1994; Grilo, Masheb, & Wilson, 2001).

#### Brief Symptom Inventory

The Brief Symptom Inventory (BSI; Derogatis, 1991) was used to assess general psychiatric functioning. The global severity index was included in validation analyses comparing women with BED exhibiting threshold overvaluation, women with BED exhibiting subthreshold overvaluation, and PCs. Scores on the BSI were converted to T-scores ranging from 0 to 100, with higher scores indicating more severe psychiatric symptoms. The BSI has good internal consistency and is highly correlated with the more lengthy Symptom-Checklist-90-R (SCL-90-R) across measured domains of psychopathology (Derogatis, 1991).

#### Social Adjustment Scale

The Social Adjustment Scale (SAS; Weissman & Bothwell, 1976) was used as a general measure of social functioning in a broad range of domains (e.g., role performance, interpersonal relationships, social and leisure activities). The SAS total score was included in validation analyses comparing women with BED reporting threshold overvaluation, women with BED reporting subthreshold overvaluation, and PCs. Scores on the SAS range from 0 to 5, with higher scores indicating poorer social functioning. The SAS has good reliability and validity (Goldman, Skodol, & Lave, 1992; Weissman, Prusoff, Thompson, Harding, & Myers, 1978).

#### Healthcare utilization

Health care utilization was determined by whether participants reported any participation in therapy/counseling in the year prior to assessment. This variable was used as a validator in analyses comparing women with BED endorsing threshold overvaluation, women with BED endorsing subthreshold overvaluation, and PCs.

### Statistical Analyses

Preliminary descriptive analyses were conducted using ANOVA and chi-square tests. These analyses included three groups: women with BED exhibiting threshold overvaluation, women with BED exhibiting subthreshold overvaluation, and PCs.

In order to determine a threshold value of shape and weight overvaluation that best predicts higher levels of eating-related psychopathology, women with BED were first categorized as more or less severe using a median split of the EDE-Q global severity index (median = 3.38). Next, the EDE-Q overvaluation of shape and weight composite variable was entered into a receiver operating characteristics (ROC) analysis to determine an optimal value for predicting membership into the more severe group. The purpose of this analysis was to establish a clinically significant threshold of shape and weight overvaluation^1^.

In order to examine the construct validity of clinical overvaluation of shape and weight, first, a MANCOVA of concurrent variables measuring current psychosocial functioning was conducted. The BSI global severity index and SAS total score were included as dependent variables in the MANCOVA, with group (BED threshold, BED subthreshold, PC) as the independent variable. The model included BMI and race as covariates, given findings that both of these variables are associated with body dissatisfaction in adults (Allaz, Bernstein, Rouget, Archinard, & Morabia, 1998; McLaren & Kuh, 2004; Wildes, Emery, & Simons, 2001). Post-hoc Tukey's honestly significant difference tests were used to examine pairwise differences in the dependent variables.

Several additional statistical tests were conducted for further validation. First, a chi-square test was used to compare the three aforementioned groups (i.e., BED threshold, BED subthreshold, and PC) on the dichotomous dependent variable of healthcare utilization in the year before assessment (i.e., whether participants did or did not participate in therapy/counseling in the previous year). Next, two individual Mann-Whitney U-tests were used to compare women with BED reporting threshold and subthreshold overvaluation on the dependent variables of EDE-measured distress and weekly binge eating frequency. PCs were not included in these analyses since they did not complete the EDE at the baseline assessment visit. Lastly, a separate chi-square test was used to compare women with BED reporting threshold and subthreshold overvaluation on the dependent variable of psychiatric comorbidity (i.e., whether participants received a comorbid SCID diagnosis). PCs were not included in these analyses since all of these participants had a psychiatric diagnosis, and thus their inclusion would have biased test results.

Finally, a discriminant function analysis was performed to determine if the threshold value of shape and weight overvaluation accurately predicts membership into the BED vs. PC group.

## Results

### Determining a Threshold of Shape and Weight Overvaluation

**Figure 1:**
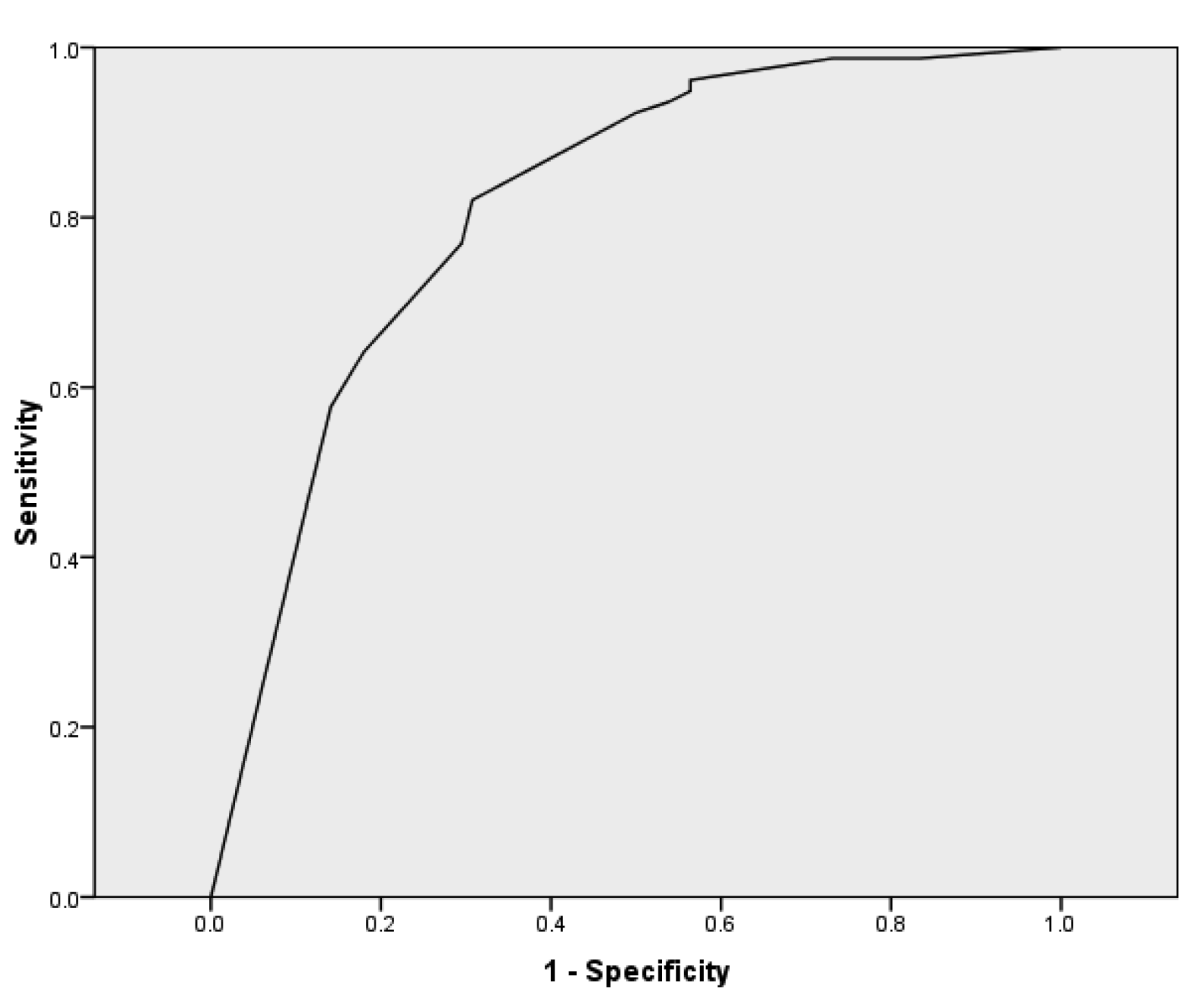
Receiver operating characteristics curve. *Note*: Receiver operating characteristics curve predicting membership to the higher severity binge eating disorder subgroup based upon shape and weight overvaluation mean score.

ROC analysis indicated that in predicting membership into the more severe BED group (i.e., those scoring above the median EDE-Q global severity index score; *n* = 78), the optimal compromise between sensitivity and specificity was achieved at a score of 4.5 on the overvaluation of shape and weight composite item (sensitivity = 0.82, specificity = 0.69; see Figure 1). This score yielded a positive predictive value of 0.73 (i.e., the proportion of individuals with threshold overvaluation who were classified as belonging to the more severe BED group) and a negative predictive value of 0.79 (i.e., the proportion of individuals with subthreshold overvaluation who were classified as belonging to the less severe BED group). A score of 4.5 indicates that shape and weight are moderately important in one's scheme for self-evaluation.

### Construct Validity of Threshold Overvaluation of Shape and Weight

#### Demographic Variables

Among women with BED, the majority (*n* = 88 out of 156; 56.4%) endorsed levels of shape and weight overvaluation at or above the threshold value of 4.5. BED subthreshold women were comprised of a significantly greater proportion of African-Americans relative to BED threshold and PC women (χ^2^(2, *N* = 260) = 25.07; *p* < .001). There were also significant group differences in BMI (*F*(2, 258) = 31.13; *p* < .001). A post-hoc Tukey's test indicated that BED threshold and subthreshold women had significantly higher BMIs than PCs, but did not significantly differ from one another. The three groups did not differ on age (*F*(2, 259) = 1.05; *p* = .35) or education level (χ^2^(4,*N* = 260) = 1.45; *p* = .84). See Table 2 for a full description of demographic characteristics.

#### Current Psychosocial Functioning

The full MANCOVA model comparing BED threshold, BED subthreshold, and PC women on measures of current psychosocial functioning was significant (*F*(2, 232) = 13.12; *p* < .001), as were univariate tests for both BSI (*F*(2, 232) = 10.05; *p* < .001) and SAS total scores (*F*(2, 232) = 10.53; *p* < . 001). Post-hoc Tukey's tests demonstrated that BED threshold women had significantly higher levels of BSI global severity than BED subthreshold (*p* < .001) and PC women (*p* < .001), whereas BED subthreshold and PC women did not differ from one another (*p* = .93). Similarly, post-hoc Tukey's tests for SAS total scores indicated that BED threshold women endorsed higher levels of interpersonal dysfunction than BED subthreshold (*p* = .002) and PC women (*p* < .001), while BED subthreshold and PC women did not significantly differ (*p* = .69).

The chi-square test for healthcare utilization indicated that BED subthreshold women were relatively less likely to have sought therapy or counseling in the year prior to assessment, compared to BED threshold and PC participants (χ^2^(2, *N* = 246) = 6.14; *p* < .05), although the standardized residual for BED subthreshold women fell within the critical values. A Mann-Whitney U-test indicated that within the BED group, women with threshold overvaluation reported greater distress over binge eating than those with subthreshold overvaluation (*Z(*156) = 4.62; *p* < .001). The two groups did not differ in frequency of binge eating episodes over the previous month (*Z(*156) = 0.65; *p* = .52). The chi-square test for psychiatric comorbidity indicated that women with BED who reported threshold overvaluation were also significantly more likely to have been diagnosed with a lifetime comorbid psychiatric disorder than women with BED who reported subthreshold overvaluation (χ^2^(1, *N* = 156) = 16.53; *p* < .001), the most common primary diagnosis being Major Depressive Disorder (*n* = 41 out of 88; 46.6%). See Table 2 for group means and test statistics.

### Predicting Diagnostic Status

Discriminant function analysis revealed that 67.7% of cases were correctly classified into their respective diagnostic group (BED vs. PC) based on the overvaluation of shape and weight cutoff score of 4.5. Specifically, among women reporting threshold levels of shape and weight overvaluation, 84.6% (*n* = 88 out of 104) were correctly classified as having BED, versus just 15.4% (*n* = 16 out of 104) who were classified as PC. Among women with subthreshold overvaluation of shape and weight, 56.4% (*n* = 88 out of 156) were correctly classified as PC, versus 43.6% (*n* = 68 out of 156) who were classified as BED. The corresponding chi-square value was highly significant (χ^2^(1, *N* = 260) = 43.76; *p* < .001).

## Discussion

The purpose of the current study was to examine the construct and discriminant validity of overvaluation of shape and weight in a large, community-based sample of women with BED and other psychiatric disorders. Threshold shape and weight overvaluation was found to be associated with impaired psychosocial functioning in women with BED, and to significantly predict diagnosis of BED versus other disorders. Taken together, these findings highlight the utility of overvaluation of shape and weight in the diagnostic scheme for BED.

Using ROC analysis, we were able to identify a value of shape and weight overvaluation that best predicted a more severely impaired profile in women with BED. Consistent with research convention (Fairburn & Cooper, 1993) and with previous studies examining this construct in BED (Grilo et al., 2008; Grilo et al., in press; Hrabosky et al., 2007; Mond et al., 2007), overvaluation of shape and weight scores corresponding to at least “moderate” importance of shape and weight in one's scheme for self-evaluation best predicted a more severely impaired subset of individuals with BED. Thus, results confirm the use of moderate overvaluation to denote clinical significance.

Women with BED who endorsed threshold overvaluation reported significantly greater levels of general psychopathology and social dysfunction than both women with BED who endorsed subthreshold overvaluation, and women with other psychiatric disorders. Moreover, among women with BED, those exhibiting threshold overvaluation reported greater levels of distress related to their binge eating, greater rates of lifetime psychiatric comorbidity, and increased healthcare utilization relative to those exhibiting subthreshold overvaluation. Threshold and subthreshold women, however, did not differ with regard to frequency of binge eating, perhaps reflecting that shape- and weight-related attitudes are less relevant triggers for binge eating episodes in BED than other psychosocial stressors (e.g., negative affect). Overall, results suggest that overvaluation of shape and weight is a clinically important construct that may be a used as a marker of impairment and distress. Grilo and colleagues (2008) have proposed that overvaluation of shape and weight may be most useful as a diagnostic *specifier* in BED (i.e., a diagnostic sub-category that can be used to aid treatment planning), rather than as a diagnostic *criterion*, the latter of which would exclude a significant portion of otherwise diagnosable patients who nevertheless experience significant impairment and distress secondary to their eating disorder. Indeed, in the current study, women with BED reporting subthreshold overvaluation exhibited commensurate levels of psychosocial impairment relative to the psychiatric control group, indicating that even without threshold levels of shape and weight overvaluation, women with BED still demonstrate marked impairment in a range of domains. Thus, current findings support including shape and weight overvaluation as a specifier rather than a criterion for the diagnosis of BED. If included as a diagnostic specifier, overvaluation of shape and weight could assist clinicians in case formulation for individuals with BED.

Discriminant function analysis revealed a modest proportion (15.4%) of PC women who endorsed threshold levels of shape and weight overvaluation. This likely reflects the relatively high levels of shape and weight concerns among women in Western societies in general (i.e., “normative discontent”; Rodin, Silberstein, & Striegel-Moore, 1985). Alternatively, even though PCs were screened for the presence of significant eating disorder symptomatology, the recruitment of a control group with psychiatric diagnoses could have inflated the rates of shape and weight overvaluation in PC women given the association between disordered eating attitudes and psychiatric disorders (Jackson & Grilo, 2002; Srebnik et al., 2003). Nevertheless, future research should explore whether shape and weight overvaluation is related to psychosocial impairment or poor treatment outcome in individuals with non-eating-related psychiatric disorders.

Strengths of this study include the large and ethnically diverse sample, and the community-based study design, which enhances generalizability of the current findings. Further, this was the first study, to the authors' knowledge, to include a psychiatric comparison group when examining shape and weight overvaluation, consequently allowing careful investigation of this variable as a clinically significant construct unique to BED. Limitations include the use of a self-report questionnaire to assess overvaluation of shape and weight. Further, PC women were not matched to women with BED on BMI, thus, some of the current findings could be attributed to increased body weight in women with BED. However, all analyses statistically controlled for BMI, minimizing such concerns.

Overall, research supports overvaluation of shape and weight as a clinically important construct in BED. Continued research on overvaluation of shape and weight in BED is warranted in order to continue to improve intervention and prevention efforts. In particular, future studies should seek to untangle timing with regards to the onset of shape and weight overvaluation relative to eating disorder and other psychosocial symptoms. Healthcare providers are advised to assess overvaluation of shape and weight in patients presenting with BED in order to obtain additional information on psychosocial functioning, and to inform clinical decision-making. For example, individuals with BED who exhibit threshold overvaluation may require interventions that address the full range of psychosocial symptoms, in addition to symptoms of BED, in order improve overall functioning and treatment outcome.

## Figures and Tables

**Table 1:** Full sample characteristics and comparisons between women with binge eating disorder and psychiatric controls on demographic variables (*M* ± *SD*, unless otherwise indicated)

Variable		Full Sample (*N* = 260)^a^	BED (*n* = 156)	PC (*n* = 104)	Test Statistic for BED vs. PC Comparison
Age, y		30.62 ± 6.19	31.04 ± 5.79	30.00 ± 6.73	*t* (258) = 1.29
Body mass index, kg/m^2^		31.01 ± 9.48	34.40 ± 9.48	25.96 ± 6.91	*t* (257) = 8.28*
	White	70.4 (183)	62.8 (98)	81.7 (85)	
Race, % (*n*)	Black	29.6 (77)	37.2 (58)	18.3 (19)	χ^2^(1, *N* = 260) = 10.71*
Education level, % (*n*)	High school or less	20.4 (53)	19.2 (30)	22.1 (23)	
	Some college	47.7 (124)	50.0 (78)	44.2 (46)	χ^2^(2, *N* = 260) = 0.85
	College graduate or higher	31.9 (83)	30.8 (48)	33.7 (35)	

* *p* ≤ .001

a Excludes 8 participants missing data on questionnaire items assessing overvaluation of shape and weight

*Note*: BED = binge eating disorder; PC = psychiatric control

**Table 2:** Demographic characteristics and psychosocial functioning of women with BED reporting threshold shape and weight overvaluation, women with BED reporting subthreshold shape and weight overvaluation, and psychiatric controls (*M* ± *SD*, unless otherwise indicated)

Variable		BED subthreshold overvaluation (*n* = 68)	BED threshold overvaluation (*n* = 88)	PC (*n* = 104)	Test Statistic
*Demographics*					
Age, y		31.37 ± 6.28	30.78 ± 5.40	30.00 ± 6.73	*F*(2, 259) = 1.05
Body mass index, kg/m^2^		35.34 ± 9.82^a^	33.66 ± 9.19^a^	25.96 ± 6.91^b^	*F*(2, 258) = 31.13**
Race, % (*n*)†	White	47.1 (32/68)^a^	75.0 (66/88)^b^	81.7 (85/104)^b^	χ^2^(2, *N* = 260) = 25.07**
	Black	52.9 (36/68)^a^	25.0 (22/88)^b^	18.3 (19/104) ^b^	
Education level, % (*n*)†	High school or less	22.1 (15/68)	17.0 (15/88)	22.1 (23/104)	χ^2^(4, *N* = 260) = 1.45
	Some college	48.5 (33/68)	51.1 (45/88)	44.2 (46/104)	
	College graduate or higher	29.4 (20/68)	31.8 (28/88)	33.7 (35/104)	

*Psychosocial functioning*					
Full MANCOVA model		---	---	---	*F*(2, 232) = 13.12**
BSI global severity index		57.56 ± 10.80^a^	64.49 ± 8.93^b^	58.18 ± 10.07^a^	*F*(2, 236) = 10.05**
SAS total score		1.97 ± 0.48^a^	2.27 ± 0.59^b^	1.90 ± 0.44^a^	*F*(2, 236) = 10.53 **
Any lifetime comorbid psychiatric diagnosis, % (*n*)†		66.2 (45/68)	92.0 (81/88)	---	χ^2^(2, *N* = 156) = 16.53**
Sought therapy/counseling in past 6 months, % (*n*)†		31.7 (19/60)^a^	51.8 (43/83)^b^	47.6 (49/103)^b^	χ^2^(2, *N* = 246) = 6.14*

*Note*: BED = binge eating disorder; PC = psychiatric control; BSI = Brief Symptom Inventory (range = 0 to 100; higher scores indicate more severe psychopathology); SAS = Social Adjustment Scale (range = 0 to 5; higher scores indicate lower social functioning). Differing superscript letters indicate significant between-group differences.

† Denominator indicates total number of available subjects

* *p* < .05

** *p* < .001

## Footnotes

1 In order to minimize concerns about the overlap between the predictor (i.e., shape and weight overvaluation composite score) and state (i.e., BED severity status based on EDE-Q global severity index median split) variables in the ROC analysis, an EDE-Q global severity index was recalculated omitting overvaluation of shape and weight items. There was a high correlation between EDE-Q global severity indices derived by including and excluding the overvaluation items (*r* = .99; *p* < .001). Moreover, ROC analyses yielded the same optimal shape and weight overvaluation score of 4.5, whether the standard EDE-Q global severity index (i.e., including shape and weight overvaluation items) or the recalculated EDE-Q global severity index (i.e., excluding shape and weight overvaluation items) was used to classify a more severe BED group.
